# Viral-mediated inflammation by Poly I:C induces the chemokine CCL5 in NK cells and its receptors CCR1 and CCR5 in microglia in the neonatal rat cerebellum

**DOI:** 10.1515/nipt-2024-0002

**Published:** 2024-04-23

**Authors:** Miguel Perez-Pouchoulen, Amanda S. Holley, Erin L. Reinl, Jonathan W. VanRyzin, Amir Mehrabani, Christie Dionisos, Muhammed Mirza, Margaret M. McCarthy

**Affiliations:** Program in Neuroscience, 12264 University of Maryland School of Medicine, Baltimore, MD, USA; Department of Pharmacology, 12264 University of Maryland School of Medicine, Baltimore, MD, USA; UM-MIND, 12264 University of Maryland School of Medicine, Baltimore, MD, USA

**Keywords:** NK cells, chemokines, microglia, vermis, development

## Abstract

**Objectives:**

To study the effect of viral inflammation induced by Polyinosinic:polycytidylic acid (PIC) on the cerebellum during a critical period of development in rats.

**Methods:**

Neonatal rat pups were treated with PIC on postnatal days (PN) 8 and 10 after which we quantified RNA using Nanostring, qRT-PCR and RNAscope and analyzed immune cells through flow cytometry and immunohistochemistry on PN11. Using the same paradigm, we also analyzed play juvenile behavior, anxiety-like behavior, motor balance using the balance beam and the rotarod assays as well as fine motor behavior using the sunflower seed opening test.

**Results:**

We determined that male and female pups treated with PIC reacted with a significant increase in CCL5, a chemotactic cytokine that attracts T-cells, eosinophils and basophils to the site of inflammation, at PN11. PIC treatment also increased the expression of two receptors for CCL5, CCR1 and CCR5 in the cerebellar vermis in both males and females at PN11. *In-situ* hybridization (RNAscope^®^) for specific transcripts revealed that microglia express both CCL5 receptors under inflammatory and non-inflammatory conditions in both males and females. PIC treatment also increased the total number of CCL5^+^ cells in the developing cerebellum which were determined to be both natural killer cells and T-cells. There were modest but significant impacts of PIC treatment on large and fine motor skills and juvenile play behavior.

**Conclusions:**

Our findings suggest an important role for CCL5 and other immune cells in mediating inflammation in the developing cerebellum that potentially impact the maturation of cerebellar neurons during a critical period of development.

## Introduction

The cerebellum was long considered exclusively involved in motor functioning, but this narrow view has expanded as substantial evidence confirms cerebellar function extends into a spectrum beyond motor control including social and emotional behaviors as well as cognitive function [1–4]. Interest increased further with discovery of a central role for the cerebellum in a range of developmental neuropsychiatric disorders [5, 6], including the observation that early life cerebellar lesions are one of the greatest risks associated with the development of autism spectrum disorders [7–9].

The developmental time frame for the cerebellum is notably long as it is one of the first brain regions to differentiate yet one of the last to fully mature. This extended window renders it particularly vulnerable to perturbations [10–13]. Significant cytoarchitectural remodeling occurs during the second postnatal week in the developing cerebellum of the laboratory rat in which there is a homeostatic increase in prostaglandin-E2 (PGE2) synthesis, peaking at around 10-days-of-age before declining at 2–3 weeks. PGE2 is a potent stimulator of the expression and activity of the estrogen synthesizing enzyme, aromatase, and there is a concomitant increase in local estradiol production during the same period [11–13]. Disruptions in either PGE2, via pharmacological inhibition of the COX enzymes, or estradiol synthesis via inhibition of aromatase, disrupts the final maturation of the Purkinje neuron (Pkn) dendritic tree [11–13]. Moreover, increased production of PGE2 due to inflammation induced by lipopolysaccharide (LPS) during the second postnatal week, also results in higher levels of estradiol in the cerebellum and stunted arborization of Pkn in both males and females, with enduring social behavior deficits, particularly juvenile playfulness only in males [11].

Here, we sought to use a model of inflammation induced by peripheral viral infection, as opposed to bacterial, via treatment with Polyinosinic:polycytidylic acid (PIC). We report that male and female rats experienced an increase in the inflammatory cytokine CCL5 and natural killer cells (NK), a type of lymphocytes of the adaptive immune system, in response to PIC. Additionally, we found that microglia express the CCL5 receptors, CCR1 and CCR5, under homeostatic conditions, but both increase in expression in response to PIC in the developing cerebellum. Microglia are considered part of the innate immune system [14–17], suggesting cross talk between the adaptive and innate immune systems in the cerebellum in response to virally-mediated peripheral inflammation. We also found a modest impairment in cerebellar-mediated loco- and fine-motor skills as well as social play behaviors after treatment with PIC.

## Materials and methods

### Experimental subjects

Sprague-Dawley rats were used in all studies and the day of birth was designated as postnatal day 0 (PN0). Animals were housed in polycarbonate cages with corncob bedding under 12:12 h reverse light/dark cycle, with *ad libitum* water and food. All procedures were approved by The Institutional Animal Care and Use Committee of the University of Maryland, Baltimore (Protocol # 0420013).

**Figure 1:**
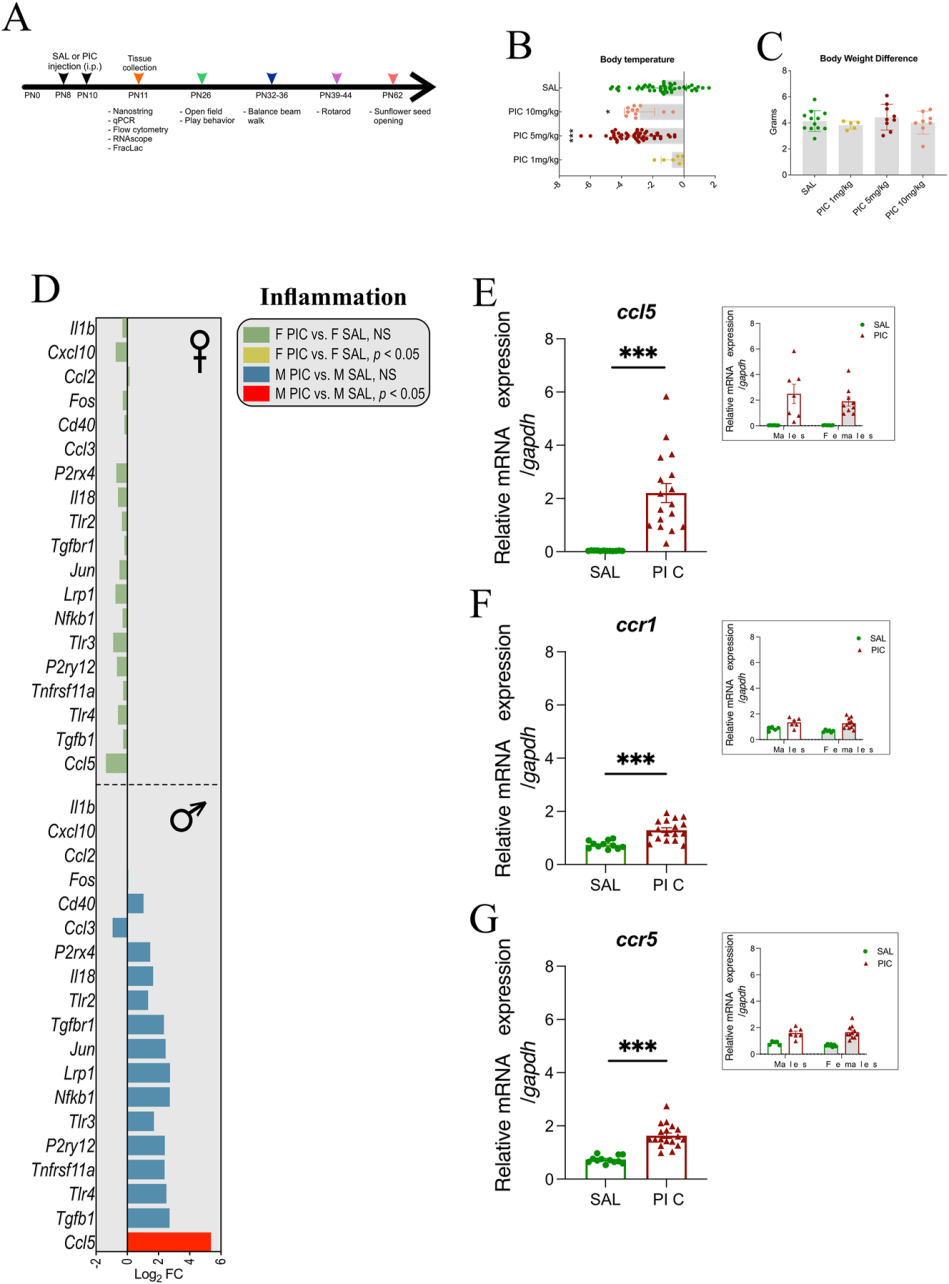
Peripheral inflammation induces expression of CCL5, CCR1 and CCR5 mRNA in the developing vermis. (A) Experimental timeline for all analysis performed. (B) A significant main effect for body temperate was found based on dose (F_(3,112)_=16.3, p<0.001), and the multiple comparison analysis showed that both 5 mg/kg of PIC (p<0.001) and 10 mg/kg (F_(3,112)_=16.3, p=0.012), but not with 1 mg/kg (p>0.05), significantly decreased compared to the SAL group. (C) No significant changes in body weight were observed in animals treated with different doses of PIC (F_(3,33)_=0.663, p=0.581). (D) NanoString analysis of mRNA revealed a significant increase of the chemokine *CCL5* only in males (red bar) after PIC treatment in the rat cerebellum at PN11. (E) qPCR of *CCL5* and its receptors *CCR1* (F) and *CCR5* (G) mRNA expression in the developing vermis at PN11 indicated a significant increase after PIC treatment in both sexes (F_(1,24)_=22.884,***p<0.001, effect size=0.488, sp=0.996). PN, postnatal day; SAL, saline; PIC, Poly I:C; inset shows data disaggregated by sex, F, female; M, male; i.p., intraperitoneal; log_2_FC, fold change; NS, not significant; sp, statistical power).

On PN8 and PN10, pups were administered an intraperitoneal (i.p.) injection of either PIC or saline (SAL). Three doses of PIC were piloted (1, 5, and 10 mg/kg). The 5 mg/kg and 10 mg/kg doses significantly reduced body temperature, but 10 mg/kg induced 50 % mortality, therefore we chose 5 mg/kg of PIC for all experiments. Body temperature was measured via rectal temperature prior to PIC injection and 3 h post injection which significantly decreased after PIC injection (Figure 1B). Bodyweight was also monitored throughout the course of treatment and there were no significant differences across groups (Figure 1C).

### qRT-PCR and NanoString

Male and female pups from each condition were deeply anesthetized and their cerebellums dissected at PN11 and PN30. The cerebellum was removed, and 1 mm thick sections taken from the vermis, sections were then flash frozen using 2-methylbutane (Sigma-Aldrich M32631) and stored at −80 °C.

For qRT-PCR, samples were suspended in Qiazol (Qiagen #79306) with zirconia/silica beads (BioSpec #11107911Oz) for tissue homogenization. Subsequently, samples were added with BAN mixed, and centrifuged to remove the clear layer from the mixture. The supernatant was mixed with 70 % ethanol and transferred into an RNeasy spin column according to protocol (Qiagen #74106/74104). RNA quality was assessed using Agilient TapeStation 4150 (samples with RNA integrity≥8 were selected). cDNA was prepared using the Transcriptor First-Strand cDNA Synthesis Kit (Roche Applied Science 04897030001) and triplicates ran for each gene of interest (QuantStudio™). Specific primers were designed using Primer Express v3.0 (Table 1).

**Table 1:** List of reagents.

Reagent	Supplier name	City	Country
Polyinosinic–polycytidylic acid sodium salt	Sigma-Aldrich P1530	St. Louis, MO 68178	USA
Saline solution	Quality Biological 114-055-101	Gaithersburg, Maryland, 20879	USA
Fatal-Plus® (NDC 0298-9373-68)	Vortech Pharmaceuticals, LTD	Dearborn, Michigan 48126	USA
2-Methylbutane	Sigma-Aldrich 270342	ST. LOUIS, MO 68178	USA
Qiazol	Qiagen QGN-79306	Germantown, MD 20874	USA
Zirconia/silica	Research Products International 9835	Mount Prospect, IL 60056	USA
BAN	Thermo Scientific 106631000, CAS 104-92-7	Headquarters in Waltham, MA	USA
RNase-free ethanol (70 %)	Fisher BioReagents BP8201-1	Fair Lawn, NJ 07410	USA
RNeasy mini kit	Qiagen QGN-74106	Germantown, MD 20874	USA
Qubit 4 Fluorometer	Thermo Fisher Q33238	Headquarters in Waltham, MA	USA
Agilient TapeStation 4150	Agilent Technologies G2992A	Linthicum, Maryland 21090-227	USA
High-Capacity cDNA Reverse Transcription kit	Applied biosystems Thermo Fisher Scientific INV-4368814	Vilnius, Lithuania (Also has location in Foster City, CA)	USA
Transcriptor First-Strand cDNA Synthesis Kit	Roche Applied Science 04897030001	Hoffmann-La Roche Inc Little Falls NJ 07424	USA
SYBR green	Applied Biosystems by Thermo Fisher Scientific 4367659	Headquarters in Waltham, MA City, CA)	USA
PCR plate	Applied Biosystems 4309848	Headquarters in Waltham, MA	USA
Mini Plate Spinner Centrifuge	ThermoFisher #14100141	Headquarters in Waltham, MA	USA
AB ViiA7	Applied Biosystems by Life Technologies 4453552	Headquarters in Waltham, MA	USA
Collagenase-D	Sigma-Aldrich 11088858001	ST. LOUIS, MO 68178	USA
DNase I	Sigma-Aldrich S10104159001	ST. LOUIS, MO 68178	USA
Percoll®	Sigma-Aldrich P4937-100ML	ST. LOUIS, MO 68178	USA
DPX	Sigma-Aldrich 06522-500ML	ST. LOUIS, MO 68178	USA
DAB	Sigma-Aldrich D5637-10G	ST. LOUIS, MO 68178	USA
PBS	Quality Biological 119-069-131	Gaithersburg, Maryland, 20879	USA
Triton	Sigma-Aldrich X100-500ML	ST. LOUIS, MO 68178	USA
Bovine Serum Albumin	Sigma-Aldrich A7906-100G	ST. LOUIS, MO 68178	USA
Paraformaldehyde	Sigma-Aldrich 441244-1KG	ST. LOUIS, MO 68178	USA
Fixable Viability Dye eFluor 780	Thermo Fisher INV-65-0865-14	Headquarters in Waltham, MA	USA
Anti-CD32	BD Biosciences 550273 mouse anti-rat	Headquarted in Franklin Lakes, NJ 07417-1180	USA
Streptavidin tags	BD Biosciences	Headquarted in Franklin Lakes, NJ 07417-1180	USA
Cytek Aurora three-laser flow cytometer	CYTEK R. 2.035	East coast USA location is Bethesda, MD 20814, this is likely where ours came from	USA
Cryostat	Microm GmbH HM550 956444	69190 Walldorf, Germany	Germany
RNAscope oven	HybEZ II, ACD 240200ACD	Headquarters in Newark California 94560	USA
special pen	ImmEdge Pen Ref. H-4000, Vector Labs	Headquartered in Newark, California 94560	USA
ProLong^TM^ Glass Antifade Mounting media	Invitrogen P36984	Eugene, OR	USA
Iba1	Wako Pure Chemical Corporation 019-19741	Osaka, Japan (Also located in Richmond, VA 23237)	USA
Triton X-100	Sigma-Aldrich X100-500ML	ST. LOUIS, MO 68178	USA
Methyl green	Sigma-Aldrich 198080-10G	ST. LOUIS, MO 68178	USA
Xylene	Sigma-Aldrich 534056-4L	ST. LOUIS, MO 68178	USA
MB-10	VWR 10121-236	US Headquarters in Radnor, PA 19087-8660	USA
Rotarod	PanLab LE8305	Holliston, MA 01746	USA

For NanoString^®^, RNA aliquots from all groups were made (5 ng/μL) and processed on the NanoString^®^ nCounter Analysis System by the University of Maryland’s Institute for Genome Sciences following a customized panel that included 171 genes out of which 33 genes were related to the immune system, eight to inflammation, 26 to phagocytosis and 10 to immune cell identity providing broad coverage of an important biological process. Results were analyzed using nSolver software (V4.0) by normalizing gene expression to the geometric mean of: (1) positive and negative controls included in the NanoString Codeset to adjust for background thresholding and to normalize sample input and differences in hybridization, and (2) the geometric mean of 10 reference genes for codeset content normalization. Significance was determined by a two-tailed *t*-test of the log-transformed normalized data [18].

### Flow cytometry

Pups were treated with PIC (i.p.) on PN8 and PN10, and on PN11 intracardially perfused with PBS. Cerebellum was dissected and blood collected and processed accordingly to Reinl et al., [19]. Cytometry was performed on a Cytek Aurora three-laser flow cytometer (CYTEK) and data were analyzed using FlowJo version X.

### RNAscope

On PN11, animals were perfused with SAL and 4 % PFA. Cerebellums were dissected and placed into 4 % PFA over night at room temperature and then in 30 % sucrose before storage at −80 °C. Cerebellums were sagittally sectioned at 45 µm with a cryostat (Microm HM550) and treated according to VanRyzin et al. 2022 using the following probes: CCL5 473641-C4, CCR1 1044031-C1, CCR5 1044041-C2. Confocal images were captured on a Nikon W-1 Spinning disk Ti2 inverted microscope (10–20x) equipped with a Hamamatsu sCMOS camera. Three cerebellar sections from the vermis were studied and the stitching tool was used to enabled visualization of the entire vermis for analysis. Additionally, Z-stack images were captured at 20x magnification using 0.2 um Z-steps to count microglia cells, deconvolved using the automatic algorithm in Nikon Elements and reconstructed in 3D using Imaris 11. Using the module Spots, we created cell markers to identify and count cells.

### FracLac analysis

We performed DAB immunohistochemistry against Iba1 (Wako 019–19741) using 45 μm cerebellar sections (for specific details, see Perez-Pouchoulen et al. 2015). Images were taken at 20x and uploaded into Image J (FIJI). Five microglia per region (3 regions × 2 sections) were randomly selected for analysis for a total of 30 cells. The selected microglia were outlined automatically by the software but manually adjusted. The plug-in *FracLac* calculated detailed morphological parameters for fractal dimension, lacunarity, density, area, circularity, and maximum radius.

### Behavioral tests

On PN26, animals were tested for open field (10 min). Total locomotion and center time were analyzed to rule out confounds due to differences in activity or anxiety-like behavior. To assess playfulness, animals were tested once a day from PN27-30 (same-sex and same-treatment partners) and video recorded for 10 min to determine number of pounces, pins, and boxing events.

### Balance beam walk

On PN32 until PN36, rats were placed, individually, on a horizontally oriented squared dowel rod (96.5 L × 2.5 W cm) suspended 12 inches above the floor. The number of foot-faults and the latency to cross the entire beam were measured.

### Rotarod

Thirty-nine day old rats were placed on a rubber rotating treadmill that gradually accelerated from 4 to 40 rpm’s during 300 s. When the animal fell, a switch stopped the automated timer and the latency per trial was measured. Animals underwent three trials per day for five consecutive days and the average latency was used to determine group differences in locomotor dexterity [20].

### Sunflower seed opening

On PN62 rats were placed into an acrylic cage (47 L × 25 W × 21.5H cm) containing four sunflower seeds (Giant brand). The time rats interacted with each seed was video scored from the first contact to the last interaction. Only trials where seeds were consumed 75 % were included in the analysis [20]. 12 hours before the test, rats were food deprived.

### Statistical analysis

All data are expressed as mean & SEM unless otherwise specified. Effect size estimate calculations (*η*) and statistical power are reported. A two-way ANOVA with treatment and sex as fixed factors was used to analyze real time qPCR, flow cytometry, RNA scope, FracLac Analysis, open field, juvenile social play behavior, balance beam walk, and sunflower seed opening data. Rotarod data was analyzed using a one-way ANOVA repeated measures. Student’s *t*-test was used to compare normalized expression values between groups and p values were adjusted using the FDR method for multiple comparisons. All other statistical analysis followed a *post hoc* pairwise comparison using the Holm’s sequential Bonferroni correction to control for familywise error. Significance was denoted when p≤0.05. All statistical tests were computed in SPSS 28 and graphed using GraphPad Prism nine.

## Results

### PIC increases CCL5 in the vermis in neonate rats

NanoString analysis indicated that CCL5 was the only gene that significantly increased in the vermis after treatment with PIC, only in males (Figure 1B). Similarly, we found that PIC significantly increased RNA levels of CCL5 in the vermis (Figure 1C, left panel). This increase was similarly detected in males and females (Figure 1C, inset), and no interaction between treatment and sex was found (Figure 1C, inset). CCL5 has several receptors and we found that mRNA for CCR1 and CCR5 increased after PIC treatment in males and females in a similar fashion in the neonate vermis (Figure 1D and E). No sex differences were detected (Figure 1D and E, see insets).

### PIC increases peripheral immune cells in the developing vermis

**Figure 2:**
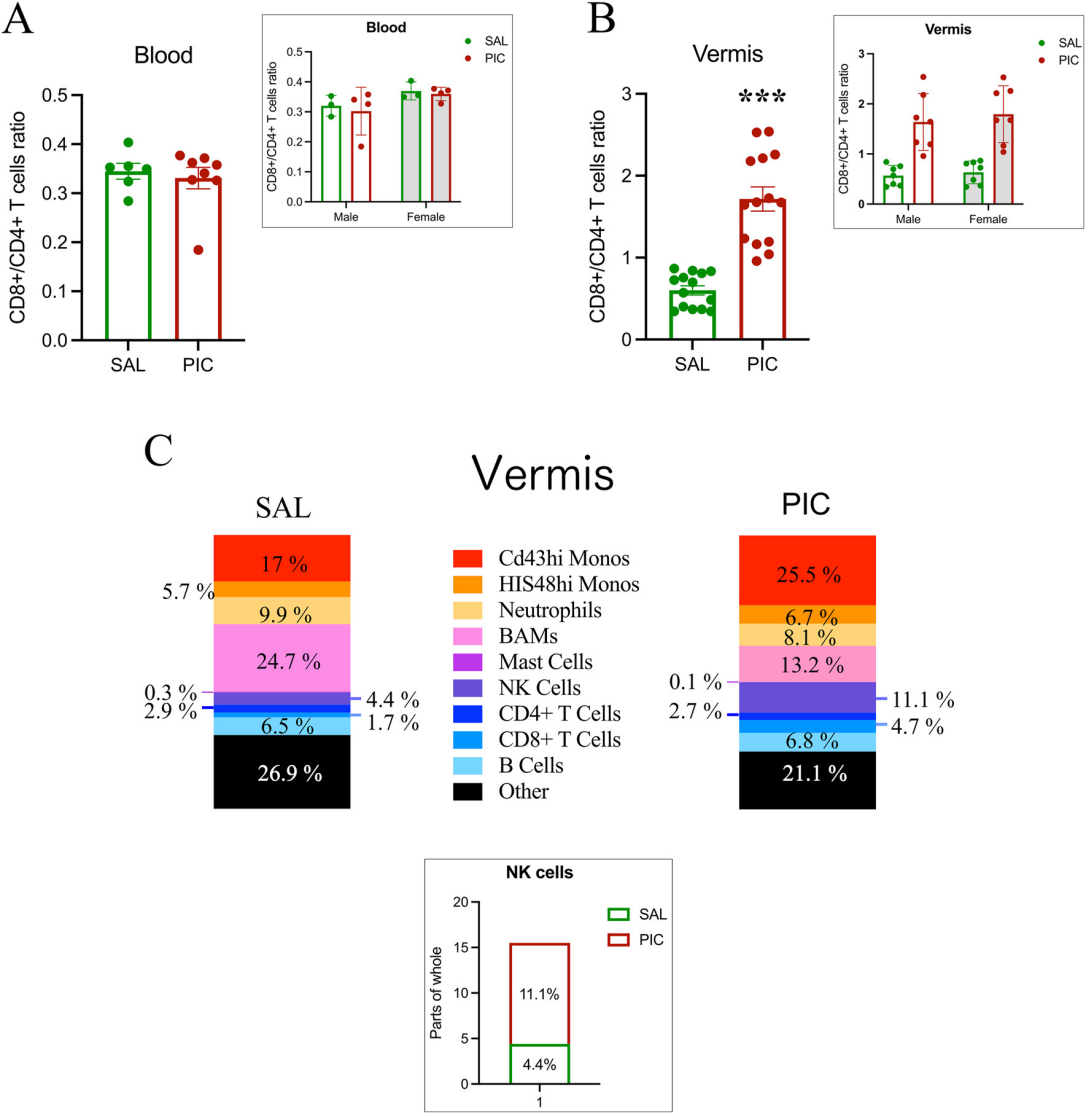
Peripheral inflammation induces immune cell influx in the developing vermis. (A) Using the same gating strategy described in Reinl et al. [19], our flow cytometric analysis of blood indicated equal CD8^+^/CD4^+^ T cell ratios in animals treated with SAL and PIC. Inset shows data disaggregated by sex. (B) In the vermis of the same animals there was a significant increase in the CD8^+^/CD4^+^ T cell ratio after PIC treatment compared to SAL controls at PN11 (*t*-test; ***p<0.001). Inset shows data disaggregated by sex. (C) The percentage of each immune cell population in the developing rat cerebellum treated with SAL (n=8) and PIC (n=6) at PN11. Inset depicts a bar representation of the percentage data shown for NK cells after treatment with SAL and PIC to highlight the difference. SAL, saline; PIC, Poly I:C; CD, cluster of differentiation; BAM’s, border-associated macrophages; NK, natural killer cells.

PIC treatment had no impact on the CD8^+^CD4^+^ cell ratio in the blood (Figure 2A) but the number of CD8^+^CD4^+^ T-cells significantly increased in the vermis after treatment with PIC (Figure 2B). No sex differences were detected in either the vermis or the blood. Exploring the proportion of white cells in the vermis after PIC treatment, we found the greatest change was in NK cells followed by the proportion of CD4^+^ and CD8^+^ T-cells (Figure 2C).

### PIC increases the number of CCL5^+^ cells in the developing vermis

**Figure 3:**
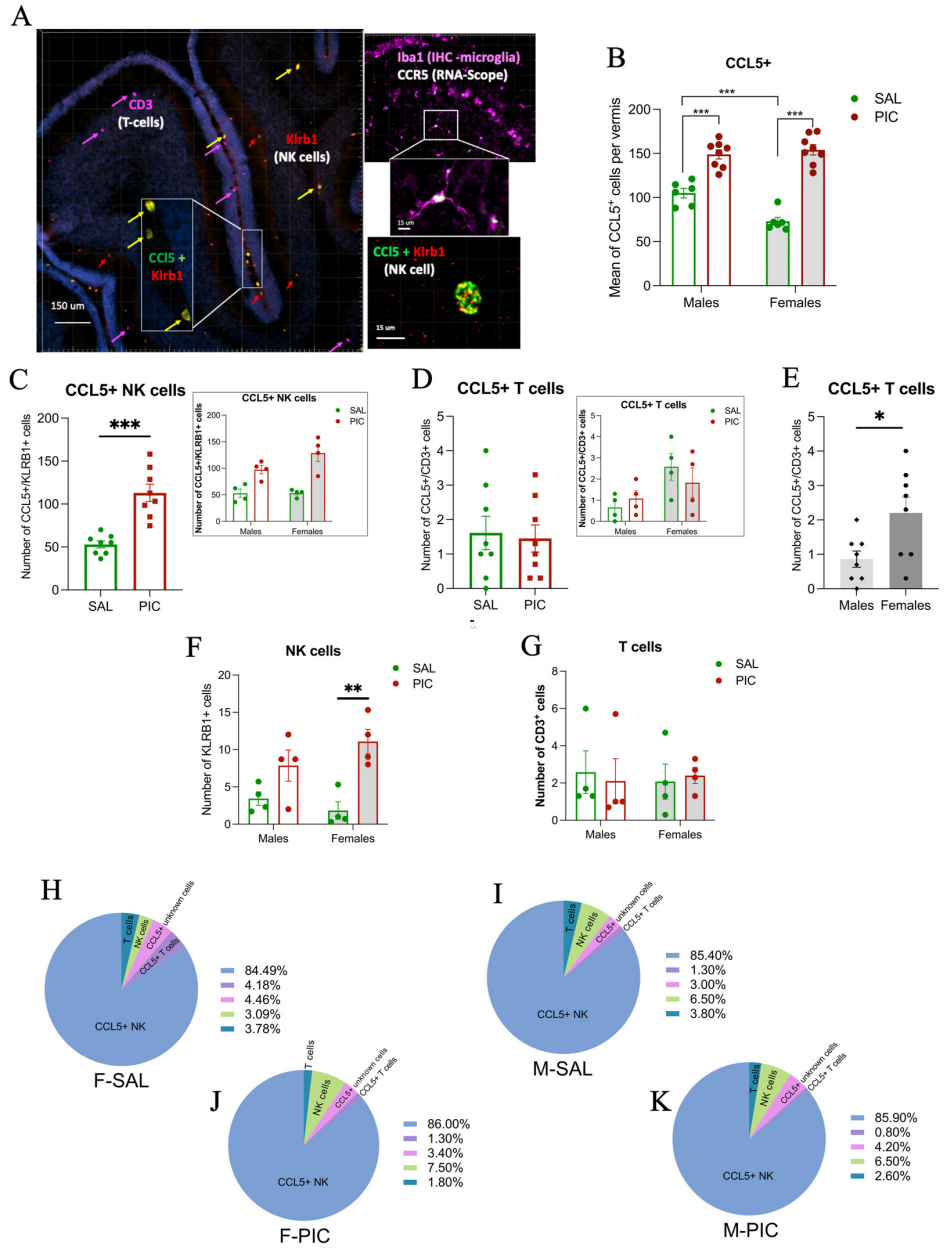
NK cells and T-cells co-localize with CCL5 but only CCL5^+^ NK cells increased after treatment with PIC in the developing vermis. (A) Confocal image of a sagittal section of the vermis showing the location of T-cells (CD3, pink), NK cells (KLRB1, orange) and CCL5^+^ NK cells (yellow) in the cerebellar parenchyma at PN11. The top-right panel presents co-localization of CCR5 and Iba1. (B) There is a significant interaction of treatment and sex as main factors (2-WAY ANOVA; F_(1, 24)_=11.459; p=0.02). Post-hoc analysis revealed a significant increase in the number of CCL5^+^ cells in males (***p<0.001) and females (***p<0.001) in the developing vermis when treated with PIC versus SAL. SAL treated males exhibited a higher number of CCL5+ cells compared to control females (***p<0.001). No sex differences were detected between the PIC treated animals (p=0.259). (C) CCL5^+^ NK cells increased following PIC treatment in the developing vermis compared to SAL treatment in both sexes (*t*-test; ***p<0.001). Inset is data disaggregated by sex. (D) There was no treatment effect on the number of CCL5^+^ T-cells in the developing vermis. (E) Independent of inflammation there were more CCL5^+^ T-cells in females than males in the vermis at PN11 (*t*-test; *p<0.05). (F) The number of NK cells was significantly increased after treatment with PIC compared to SAL (***p<0.001) with no significant interaction of treatment and sex (inset). (G) The number of T-cells observed after treatment with PIC was similar to SAL treatment in both males and females. Pie charts in panels H, I, J, K, depict the percentage of NK cells and T-cells colocalizing and non-colocalizing with CCL5. NK, natural killer cells; CD, cluster of differentiation; KLRB1, killer cell lectin like receptor B1.

Using RNA scope, we identified CCL5^+^ cells across the cerebellar cortex (Figure 3A). Cells expressing CCL5^+^ increased in number after PIC treatment in both males and females in the vermis (Figure 3B) and were identified as NK and T-cells (Figure 3A, right insets). Treatment with PIC significantly increased the number of NK cells expressing CCL5^+^ compared to SAL (Figure 3C), but the number of T-cells expressing CCL5^+^ remained the same (Figure 3D). The number of T-cells expressing CCL5^+^ was higher in females than males, but no significant differences were detected for the interaction of treatment and sex for NK or T-cells expressing CCL5^+^ (inset in Figure 3C and D). Based on RNA scope, females exhibited a higher number of T-cells expressing CCL5^+^ compared to males (Figure 3E). A group of CCL5^+^ cells did not colocalize with the cell markers used in this experiment and no statistical differences were found (Figure 3F). A group of NK cells did not colocalize with CCL5 and they increased in number after PIC treatment compared to SAL (Figure 3G). In contrast, T-cells not expressing CCL5 were not different between groups (Figure 3H).

### PIC treatment induces CCR1 and CCR5 expression in the developing vermis

**Figure 4:**
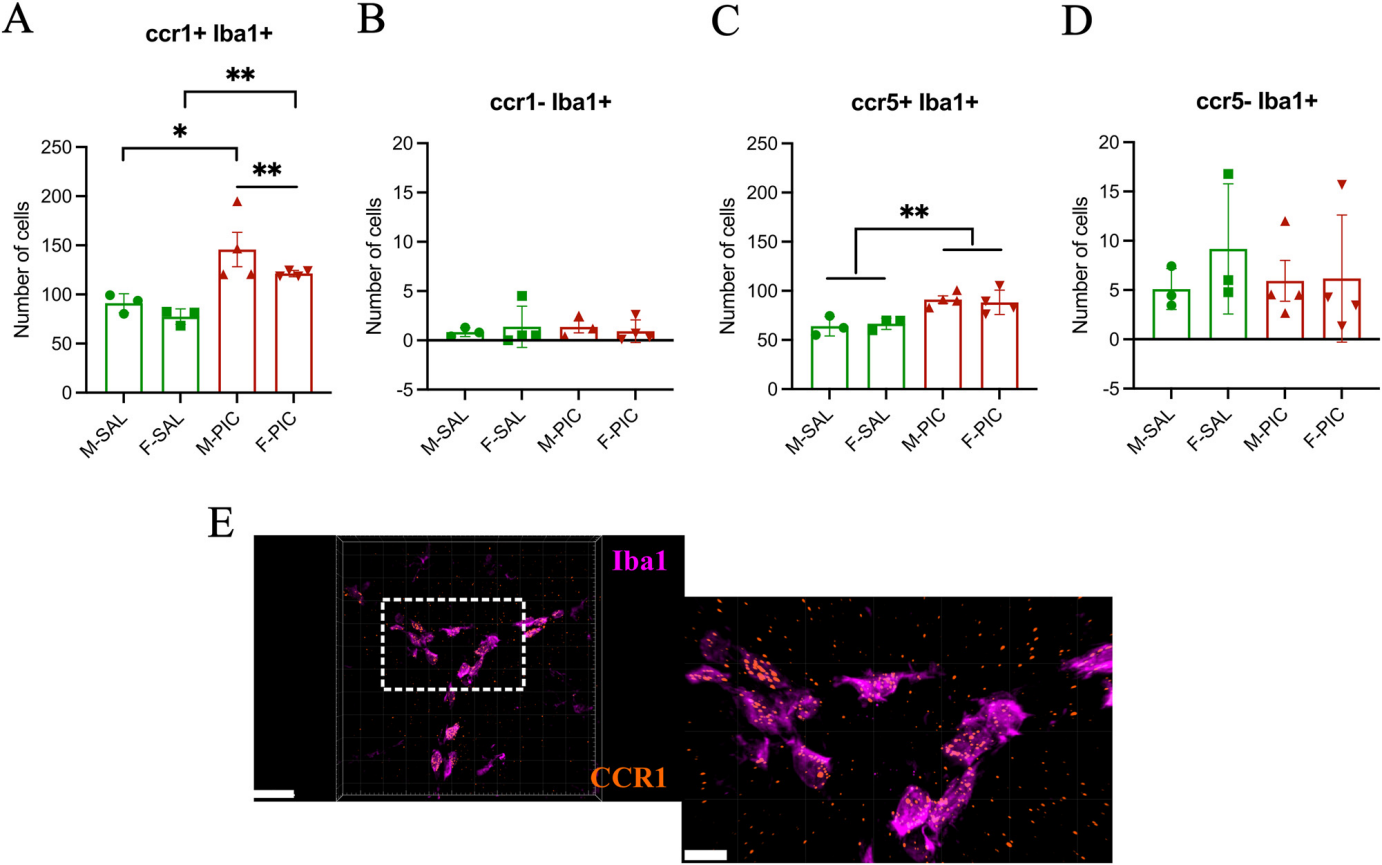
CCR1 and CCR5 are expressed by microglia and are upregulated by peripheral inflammation in the developing cerebellum. (A) Both males (*p<0.05) and females (**p<0.01) treated with PIC exhibited a higher number of CCR1^+^ microglia cells compared to SAL treated controls (two-way ANOVA, F_(1,10)_=25.014, p<0.001, effect size=0.714, sp=0.994), and this response is greater in males than females (**p<0.01). (B) The number of Iba1^+^ microglia not expressing CCR1 did not differ by treatment or sex. (C) However, more Iba1+ microglia expressed CCR5 after PIC treatment in both sexes compared to SAL controls (2-way ANOVA, [F_(1,10)_=23.368, effect size=0.700, sp=0.991]; **p<0.01). (D) The number of Iba1^+^ microglia not expressing CCR5 did not differ by sex or treatment. (E) confocal images after RNAscope analysis demonstrating co-localization of CCR1 (orange) and Iba1 (magenta) in the developing vermis at PN11. Scale bars=30 µm (left image) and 10 µm (right image).

There was a strong co-localization of microglia with CCL5 receptors CCR1 and CCR5 (Figure 3A and 4E). Treatment with PIC increased the number of microglia expressing CCR1 in both males and females (Figure 4A). In contrast, no effect of PIC treatment was observed in microglia that did not express CCR1 (Figure 4B). Similar results were detected for microglia expressing CCR5 (Figure 4C) and microglia lacking CCR5 (Figure 4D).

### Microglia morphology remains unaffected after treatment with PIC

**Figure 5:**
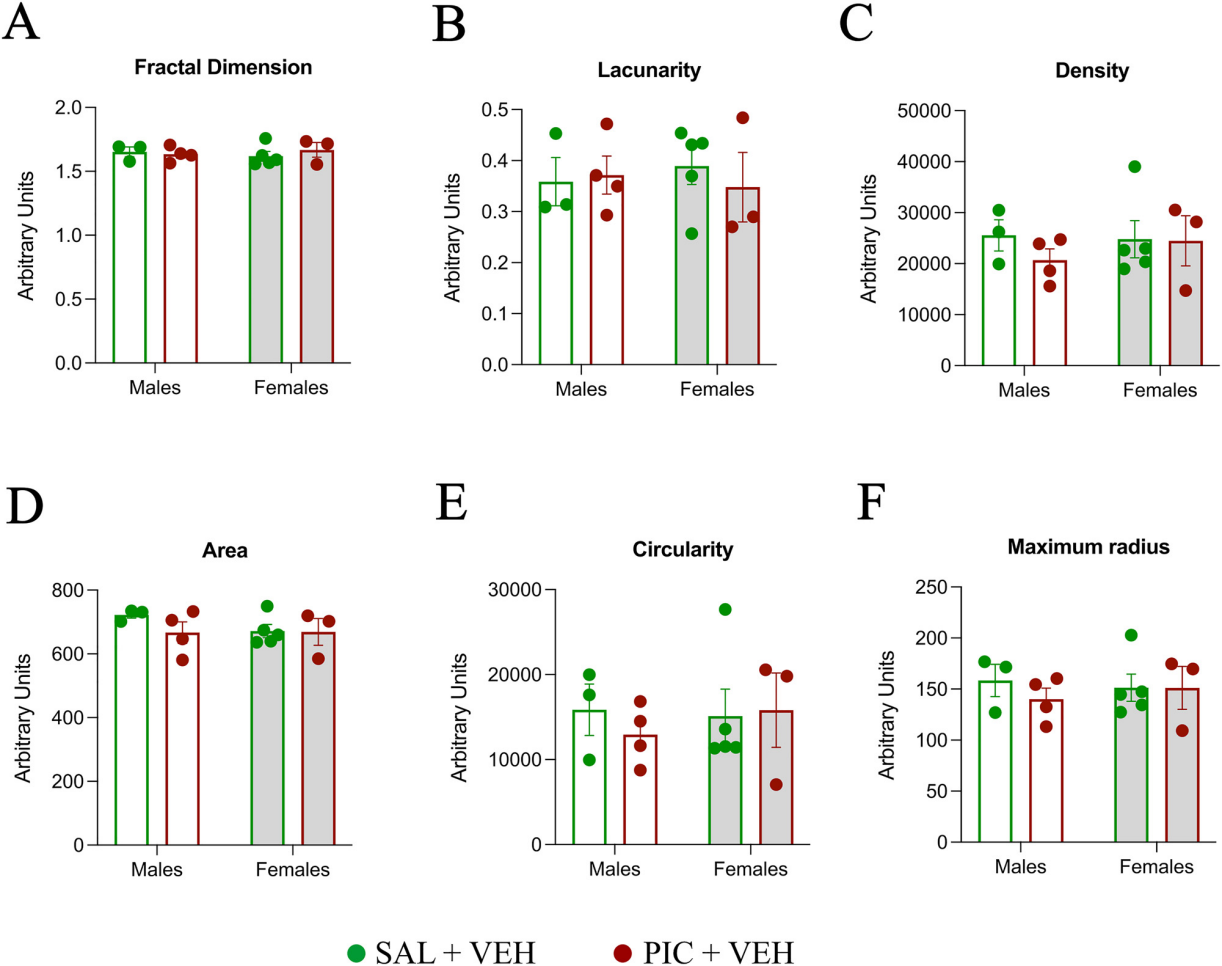
FracLac analysis of microglia morphology after treatment with PIC. There were no significant differences for the parameters of fractal dimensions (A), lacunarity (B), density (C), area (D), circularity (E) or maximum radius (F) in the PN11 vermis in males and females.

We investigated the effect of PIC on microglia morphology in the vermis using FracLac analysis and detected no morphological changes for the Fractal Dimensions, Lacunarity, Density, Area, Circularity and Maximum radius when treatment was the main factor (Figure 5). No sex differences were detected either nor any significant interactions of treatment and sex.

### No anxiety-like behaviors were observed in rats treated with PIC

**Figure 6:**
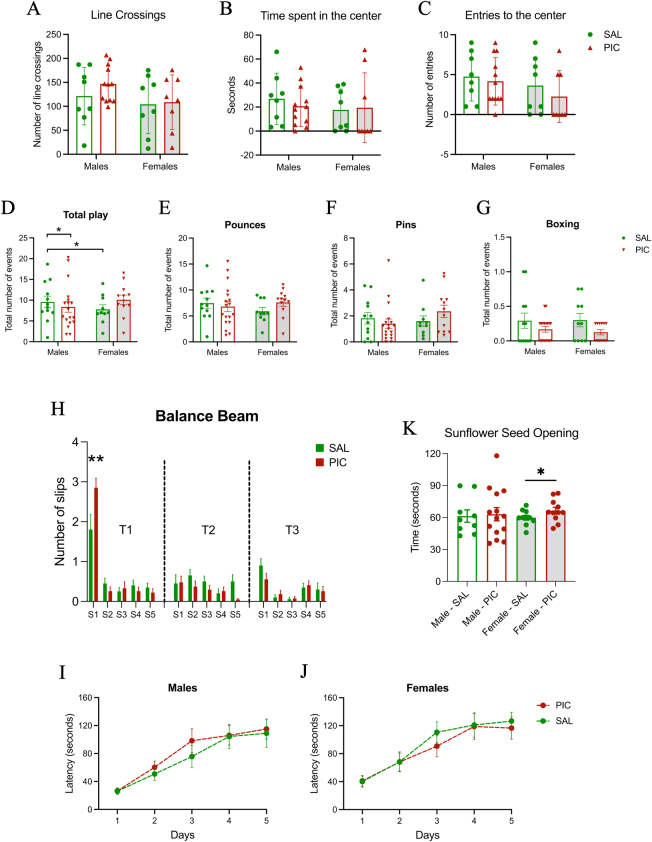
Social and motor behavior effects of peripheral inflammation in the juvenile and adult rat. Open field testing of animals treated with PIC or SAL detected no differences in (A) line crossing, (B) time spent in the center and (C) entries to the center at PN26. (D) Analysis of social play behavior indicated that males treated with PIC were less playful than control males when all play events were totaled (F_(1, 45)_=6.519, effect size=0.127, sp=0.705; *p=0.014). As expected, control males played more than control females. When play was broken down into individual components no single component was significantly affected by PIC treatment. These include (E) pounces (F), pins or (G) boxing events at PN32-36. (H) A fine motor coordination deficit was detected in animals treated with PIC when performing the balance beam test on the first trial but not in subsequent trials (main effect of treatment 2-WAY ANOVA; F_(1,43)_=5.833; *p=0.02 followed by a multiple comparison *post hoc* with the Bonferroni correction **p=0.009). Performance on the rotarod was not affected by treatment in (I) males or (J) females. (K) Females treated with PIC took longer to open sunflower seeds compared to females treated with SAL (*p=0.058, t_(1,19)_=1.647, Cohen’s d=9.06), a test of fine motor skills.

The two-way ANOVA analysis used in the open field test showed no significant differences in rats treated with PIC for the number of line crosses, the time spent in the center of the chamber or the number of entries to the center (Figure 6A).

### Play behavior remained typical in male and female rats after treatment with PIC

There were no significant effects of PIC on classical play behavior parameters such as pounces, pins, or total play events when treatment was considered the main factor (Figure 6D). Overall, playfulness was slightly but significantly reduced in males with PIC (Figure 6D).

### PIC treatment of neonatal rats temporarily impaired motor balance in late adolescents

The balance beam test found a significant effect of PIC on motor balance on the first day of the test (Figure 6H). Rats treated with PIC on PN8 and PN10 were tested beginning on PN32 and slipped more than rats treated with SAL. However, this effect was observed only during the first session on the first testing day out of 15 tests across 3 days. No sex differences were found at any phase of the experiment.

### Neonatal PIC treatment altered fine motor behavior in female rats

The sunflower seed opening test showed that females treated with PIC spent significantly more time opening the sunflower seeds compared to control (Figure 6K). There were no significant effects in males treated with PIC.

### General motor coordination in rats remained unaffected by PIC

Male and female rats treated with PIC exhibited no motor coordination abnormalities when performing the rotarod test on any trial during five consecutive days (Figure 6I and J, respectively).

## Discussion

The cerebellum is particularly vulnerable to external insults, such as peripheral inflammation, during postnatal development when it undergoes significant growth and maturation. The cerebellum is largely comprised of repeating units consisting of a Purkinje cell and its single climbing fiber from the inferior olivary nucleus, parallel fibers from granule cells and axonal output to the deep cerebellar nuclei. Pkn’s are the final common integrator of all information coming from the climbing fibers, interneurons and parallel fibers which represent the largest synaptic input of the brain with up to 200,000 synapses per cell in rodents [21]. Developmentally, each Purkinje cell is innervated by multiple climbing fibers and by a process of activity dependent competition must reduce them to one for proper function, a process largely completed during the second postnatal week [22, 23], a time coincident with the sensitive period we previously uncovered [11] and further explore here.

Abnormalities of the cerebellum are found in individuals diagnosed with autism spectrum disorders or schizophrenia [9, 24], [25], [26]. Both disorders are notable for two things; 1) the strong gender bias, age of onset and symptomology and, 2) the increased risk for diagnosis associated with gestational and early life inflammation [27–36]. Despite this, how inflammation impacts the developing cerebellum receives little attention. Evidence of inflammation within the cerebellum is seen in severe motor impairment, headaches, or neurological symptoms. Reliance on symptoms such as ataxia and complaints of headaches will not detect inflammation in infants not yet walking and talking, and lesser degrees of inflammation-mediated damage would also go undetected while nonetheless permanently altering the developmental trajectory.

PIC is a viral mimetic acting as an agonist at the TLR-3 receptor and as a neurostimulant is one of the most potent inducers of interferon, including in the pregnant dam. Treatment of pregnant dams with PIC indues interferon and disrupts fetal brain development [37, 38]. Here we focused on the postnatal pup at a time analogous to birth and the early infant period. Using an unbiased method for detection of gene expression (NanoString^®^) in a custom designed array that included 170 genes, over 50 of which are involved in immunity or inflammation, we found only one significant change in response to PolyI:C, an increase in the chemokine CCL5, and only in males. Note that we did not include interferon in our panel as it is a well established response to PIC. However, interferon directly stimulates CCL5 transcription in macrophages [39] and is therefore a likely mechanism by which PIC stimulates CCL5 production in the developing brain, although this remains to be determined. We subsequently validated by qPCR an increase in CCL5 mRNA in cerebelli of both males and females after PIC treatment and the number of CCL5^+^ cells detected by RNA-Scope^®^ also increased equally in males and females, suggesting both sexes respond to viral infection with an increase in CCL5. Also known as RANTES, CCL5 is a member of a complex family of around 50 ligands and 20 GPCRs [40]. As a chemokine CCL5 is a broad spectrum recruiter, able to attract T-cells, dendritic cells, NK cells, eosinophils, mast cells and basophils [41]. It is also produced by a large variety of cells including platelets, macrophages, endothelium and epithelium. There are at least three receptors for CCL5, CCR1, CCR3 and CCR5, as well as decoy receptors that appear to act as scavengers. This broad biological footprint reflects the myriad of functions of CCL5 in both health and disease and its impact far beyond the traditional role of a chemokine. Roles for CCL5 in the brain are still being established but include neuroprotection against excitotoxic glutamate, beta-amyloid and HIV proteins. In mouse models of prodromal Huntington’s Disease and tauopathies such as Alzheimer’s, CCL5 expression is increased and inhibits autophagy that normally degrades aggregating proteins [42]. In the adult rat and mouse, CCL5 is lowly and diffusely expressed in neurons, astrocytes and oligodendrocytes [43], in contrast to our observation that developmentally, CCL5 is restricted to immune cells, at least in the cerebellum. This may reflect a developmental phenomenon, or specificity to the cerebellum which was not examined in the above cited studies. Further complexity in CCL5 effects are found in differential signaling based on levels and duration of exposure [40].

In this study, we identified a novel involvement of NK cells and CCL5 in the inflammatory response of the developing cerebellum to the viral mimetic PIC. Interestingly, both NK cells and T-cells produce CCL5 under non-inflammatory conditions in the cerebellar vermis, while PIC significantly induced an influx of NK cells, but not T-cells, expressing CCL5. NK cells are lymphocytes of the innate immune system that are pivotal to the defense against pathogens, such as viruses, and in certain diseases like Multiple Sclerosis [44]. They also participate in healthy tissue remodeling [45] and their function is often determined by the tissue in which they reside when not in the blood stream, a process still being understood. NK cells secrete several cytokines and chemokines that can modulate the function of other innate and adaptive immune cells [46, 47]. In this instance, NK cells expressing CCL5 were found across the entire cerebellar parenchyma including the grey and white matter. T-cells are also lymphocytes and cooperate and interact with NK cells as well as microglia to thwart CNS viral infection [48], among other functions. We examined a wide range of peripheral immune cells after PIC exposure and NK cells markedly increased in the cerebellum compared to other peripheral immune cells.

The mRNA for receptors CCR1 and CCR5 also increased following PIC treatment in both sexes and were also exclusively limited to microglia. Microglia are the innate immune cells of the brain and essential to healthy development but also central to dysregulation following neuroinflammation [49–52]. Principle microglia functions include synaptic pruning [53, 54], promotion of synapse formation [55] and cell elimination [56, 57]. All of these functions can differ in developing males and females and lead to both the establishment of healthy sex differences and be the source of differential vulnerability to inflammation or injury [58, 59]. Microglia are responsive to CCL5 signaling [60] and protect the blood:brain:barrier early in peripheral inflammation but can lead to damage during sustained inflammation [61]. Microglia in the cerebellum undergo drastic morphological changes during postnatal development that are associated to their function [62]. Unexpectedly, we found that PIC had no evident impact on the morphology of microglia in the developing cerebellum. Nonetheless, the simultaneous increase in NK- and T-cells, up regulation of CCL5 by NK cells and increased expression of CCR1 and CCR5 receptors by microglia indicate a coordinated response across the innate and adaptive immune systems in response to peripheral viral infection.

To determine functional impacts of peripheral viral infection, we performed a series of behavioral tests associated with cerebellar function previously studied in our lab [11, 12]. Treatment with PIC during the second postnatal week induced an initial impairment on the balance beam when animals were tested at just over a month of age but recovered on the next trial. Given the importance of balance it is likely that other brain regions involved in motor learning compensated for the cerebellar damage after the first trial, or that the cerebellum itself adapted quickly with only brief experience. There was no impairment on the Rotorod^®^ test, suggesting gross locomotion and balance were unimpaired. The ability to open a sunflower seed is a measure of fine motor skills associated with the cerebellum. We detected a small but significantly longer latency in females exposed to PIC in opening the seed as full adults, suggesting some degree of lasting cerebellar damage. Lastly, overall playfulness reduction in males exposed to PIC earlier in life is consistent with previous impairments we have seen in male playfulness when mimicking a bacterial infection and that we associated with damage to Purkinje neurons [11–13].

Overall, our study provides novel evidence of an NK cell/CCL5 mediated response to peripheral viral infection during a postnatal sensitive period of the developing cerebellum. We detected modest but enduring impacts on behaviors previously associated with cerebellar damage, some of which were evident in only one sex and others in both sexes. Future studies should include additional morphological and functional characterization as early life inflammation is a known risk factor for neurological and developmental disorders but the mechanisms mediating that risk remains largely unknown.
